# Circulating myeloid populations have prognostic utility in alcohol-related liver disease

**DOI:** 10.3389/fimmu.2024.1330536

**Published:** 2024-03-13

**Authors:** Reenam Khan, Shees Salman, Laura Harford, Lozan Sheriff, Jon Hazeldine, Neil Rajoriya, Philip N. Newsome, Patricia F. Lalor

**Affiliations:** ^1^ Centre for Liver and Gastrointestinal Research, Institute of Immunology and Immunotherapy, Birmingham, United Kingdom; ^2^ The Liver Unit, Queen Elizabeth Hospital Birmingham, Birmingham, United Kingdom; ^3^ Institute of Inflammation and Ageing, University of Birmingham, and Birmingham National Institute for Health Research (NIHR), Biomedical Research Centre, Birmingham, United Kingdom

**Keywords:** cirrhosis, neutrophil, monocyte, alcohol, hepatitis, human

## Abstract

**Introduction:**

Alcohol-related liver disease (ARLD) accounts for over one third of all deaths from liver conditions, and mortality from alcohol-related liver disease has increased nearly five-fold over the last 30 years. Severe alcohol-related hepatitis almost always occurs in patients with a background of chronic liver disease with extensive fibrosis or cirrhosis, can precipitate ‘acute on chronic’ liver failure and has a high short-term mortality. Patients with alcohol-related liver disease have impaired immune responses, and increased susceptibility to infections, thus prompt diagnosis of infection and careful patient management is required. The identification of early and non-invasive diagnostic and prognostic biomarkers in ARLD remains an unresolved challenge. Easily calculated predictors of infection and mortality are required for use in patients who often exhibit variable symptoms and disease severity and may not always present in a specialized gastroenterology unit.

**Methods:**

We have used a simple haematological analyser to rapidly measure circulating myeloid cell parameters across the ARLD spectrum.

**Results and Discussion:**

We demonstrate for the first time that immature granulocyte (IG) counts correlate with markers of disease severity, and our data suggests that elevated counts are associated with increased short-term mortality and risk of infection. Other myeloid populations such as eosinophils and basophils also show promise. Thus IG count has the potential to serve alongside established markers such as neutrophil: lymphocyte ratio as a simply calculated predictor of mortality and risk of infectious complications in patients with alcohol-related hepatitis. This would allow identification of patients who may require more intensive management.

## 1 Introduction

It has been estimated that on a global scale, approximately half of the population currently drinks alcohol (1) with excess consumption a significant driver of disability and mortality. Alcohol-related liver disease (ARLD) accounts for over one third of all deaths from liver conditions (2), and mortality from alcohol-related liver disease has increased by 450% over the last 30 years (3). ARLD encompasses a spectrum that includes steatosis, steatohepatitis, fibrosis, cirrhosis, and subsequently hepatocellular carcinoma. Most individuals consuming >60g a day of alcohol will develop some degree of hepatic steatosis. A minority of these patients may develop the significant acute inflammatory condition called alcohol-related hepatitis (AH), and approximately 10-20% progress to liver cirrhosis (4). The presence of cirrhosis increases mortality risk by up to 10 fold (3), and much of the disease burden impacts people of working age (5). Thus, the economic cost of managing patients with ARLD is significant and growing.

Severe AH almost always occurs in patients with a background of chronic liver disease, with extensive fibrosis or cirrhosis. Severe AH can precipitate ‘acute on chronic’ liver failure (ACLF), associated with a multi-organ failure and a high short-term mortality (6). In patients hospitalized for alcohol-related cirrhosis, ACLF was associated with a three-month mortality of 60% vs 20% in patients in chronic decompensated cirrhosis (7). The most common causes of death in patients with AH include infections, GI bleeding, hepatic encephalopathy and hepatorenal syndrome. Sepsis is the most significant challenge in patients with AH with estimates suggesting that up to 25% of patients with AH have an infection on admission to hospital (8). Patients with AH have impaired immune responses, which increases their susceptibility to infections and often exhibit features of the systemic inflammatory response syndrome even in the absence of infection. This presents a challenge in the prompt diagnosis of infection and patient management. Patients with cirrhosis too exhibit immune dysfunction (9, 10) and this contributes to their overall poor outcome (11). The identification of early and non-invasive diagnostic and prognostic biomarkers in ARLD remains an unresolved challenge. In the absence of validated specific disease biomarkers, a definitive diagnosis of AH requires a biopsy (12) which carries risk in patients with compromised hepatic function. Scoring systems such as MELD and CLIF-C (13) can be used to identify at risk cirrhotic patients who need more intensive monitoring. However robust and easily calculated predictors of infection and mortality are required for use in patients who often exhibit variable presentation and disease severity and may not always present in a specialized gastroenterology unit.

Myeloid cells differentiate from bone marrow precursors to become mature circulating granulocytes (neutrophils, eosinophils, and basophils) and monocytes. These cells often function as a first line of defense against infection but can also precipitate end organ injury or drive resolution depending on the specific disease context (14). Such myeloid cells have been associated with the pathophysiology of ARLD. For example a high systemic neutrophil: lymphocyte ratio has been shown to be predictive of mortality (15), and acute kidney injury and infection (16) in the context of alcohol-related hepatitis. Similarly, recruitment of monocytes into the damaged liver contributes to inflammation and fibrosis in alcohol-related liver disease (17). Systemic inflammation or infection causes bone marrow stimulation and alterations in systemic myeloid cell counts and maturity (18). Studies suggest that elevated immature granulocyte (IG) counts may be useful as early markers of sepsis or inflammation (6, 7), although this has not been investigated in ARLD. Therefore, in this study, we use the Sysmex XN-1000 haematology analyser to investigate whether assessment of circulating myeloid cell populations also has potential prognostic utility in alcohol-related liver disease.

## 2 Materials and methods

### 2.1 Patient bloods and data collection

We collected peripheral blood from patients with alcohol-related hepatitis, (defined by ongoing alcohol use prior to admission, bilirubin >80mmol/L). We excluded patients if they had untreated sepsis, serum creatinine >500umol/L, malignancy, hepatitis B or C, or HCC or biliary obstruction. We also recruited patients with established chronic liver disease from hepatology outpatient clinics, including patients that were actively drinking and those that were abstinent >6 months. All samples were collected with informed patient consent and local LREC approval (Wales LREC reference 18-WA-0214). Healthy volunteers were also used as a control cohort. Patients were characterized into the following groups: Abstinent patients with chronic alcohol related liver disease (ARLD-Abstinent), and patients attending high dependency clinics who had alcohol-related cirrhosis and were still drinking (ARLD drinker). Demographic and clinical information was collated from digital healthcare records. Mortality outcomes were recorded up to 12 months after initial hospital admission for AH, and the presence of infection/use of antibiotics was reviewed up to 6 months post initial admittance. We supply the demographic information for our cohorts in **Tables 1**, **2**. Whole blood was analyzed on a Sysmex XN-1000 analyzer according to manufacturer’s protocols.

**Table 1:** Combined clinical characteristics of our cohorts.

Parameter (and reference ranges)	Healthy controls (n=39)	ARLD (Abstinent) (n=39)	ARLD (Drinking) (n=11)	Alcohol-related Hepatitis (n=57)
Age	34 (27-41)	58 (36-77)	61 (42-72)	58 (20-65)
Female (%)	62	46	36	38
Alcohol units consumed per week		0	37.8 (15-75.6)	160 (21-420) [data available for 55 cases]
ALT (U/L) **Reference 0 - 55**		22 (10-61)****	27 (17-44)*	54 (15-204) [data available for 53 cases]
AST (U/L) **5 - 34**		27 (15-58)****	42 (24-91)***	130.9 (38-307) [data available for 46 cases]
ALP (U/L) **30 - 130**		106 (46-307)****	135 (64-227)	190.1 (92-562)
Urea (mmol/L) **2.5 – 7.8**		6.1 (2.1-20.8)*	5.6 (1.1-37)	5.2 (1.1-37)
Bilirubin (μmol/L) **< 21**		32 (5-205)****	18 (9-38)****	248 (51-732)
Creatinine (μmol/L) **64 - 104**		73 (41-156)***	73 (43-103)	69 (31-427)
Albumin (g/L) **35 - 50**		38 (21-45)****	38 (30-52)****	23 (12-45)
INR **0.8 – 1.2**		1.06 (1.0-1.9)****	1.1 (1-1.5)****	1.76 (1-3.2)
CRP (mg/L) **0 - 5**		4 (1-37)****	7 (1-18)**	43.29 (1-136)
FIB-4 points		2.4 (0.7-10.45)**** [data available for 33 cases]	3.4 (1.5-11.5)** [data available for 10 cases]	8.3 (2.4-49.7) [data available for 35 cases]
Encephalopathy (%)		7/39 (18)	1/11 (9)	12/57 (21%)
MELD score		8 (6-26)**** 18% MELD>15	9 (6-13)**** 0% MELD>15	24 (12-69) 95% MELD>15
UKELD score		50 (43-62)****	50(46-55)****	61 (51-72)
Child Pugh Score (points)		6 (5-11)****	6 (5-7)****	11 (6-14)
GAHS score		n/a	n/a	8 (5-11)
Maddrey score		n/a	n/a	54 (5.3-137) [data available for 40 cases]
1-month mortality (%)		0/39 (0)	0/11 (0)	10/56 (18%) [1 patient excluded as data not known]
6- month mortality (%)		2/36 (6) [3 patients excluded as data unknown]	0/11 (0)	12/43 (28%) [3 patients excluded as data not known]
12-month mortality (%)		2/23 (9) [16 patients excluded as data unknown]	0/7 (0) [4 patients excluded as data unknown]	16/35 (46%) [8 patients excluded as data not known]

Table summarizing the clinical data for all patients (used in functional assays and/or sysmex analyses). Peripheral blood samples were obtained from healthy controls (HC), and patients in indicated groups. Where appropriate, reference ranges are indicated in bold in the first column. Abstinent patients had ARLD and were on our transplant assessment list whilst those who were still drinking were being seen as outpatients in our high dependency clinic. Biochemistry data, patient symptoms and medication history were obtained from University Hospitals Birmingham electronic patient records. Age is rounded to the nearest year and mortality outcomes were assessed up to 12 months after hospital admission or sampling. Data is listed as mean (min, max values). Data were reviewed for normality (using the Shapiro Wilkes Test), and depending on the result, they were analysed using a one way ANOVA or Kruskal-Wallace test with Dunns multiple comparisons *p0.05, **p<0.001, *** p<0.005, ****p<0.0001 vs Alcoholic hepatitis cohort. ALT, alanine aminotransferase; AST aspartate aminotransferase; ALP, alkaline phosphatase; INR, international normalised ratio; MELD, Model for End-Stage Liver Disease; UKELD, United Kingdom Model for End-Stage Liver Disease; GAHS, Glasgow Alcoholic Hepatitis Score; Maddrey Score, Maddrey discriminant function).

**Table 2:** Infectious consequences in patients with ALD cirrhosis (abstinent and drinkers) and AH.

Parameter	ARLD-A (n=39)	ARLD-D (n=11)	AH (n=37)
Positive blood culture	0/39	0/11	1/36 (3) [1 patient excluded as data not known]
Microbial growth in urine (%)	0/39	0/11	1/37 [enterococcus faecalis]
Presence of CXR changes to support a diagnosis of pneumonia	0/39	0/11	12/37 [n=7 consolidation, n=5 atelectasis +/- pleural effusion
Bacterial peritonitis on ascitic tap	0/39	0/11	4/37 [n=1 had growth of Clostridium Tertium, n=1 had growth of Enterococcus Cloacae, n=2 had a polymorph count >250 but no bacterial growth]
Infection on skin swabs	0/39	0/11	2/37 [n=1 patient had fournier’s gangrene, with multiple organisms on swab, one patient had growth of Staph Aureus]
Infection on nasal/throat swabs	0/39	0/11	2/37 [n=2 tested positive for COVID19]
Infection on stool culture	0/39	0/11	1/37 [n=1 patient had C diff+ve stools]
Antibiotics (during hospital admission)	0/39	0/11	26/37 [16/26 were on antibiotics at the time of blood sampling]

Electronic patient records at University Hospitals Birmingham were reviewed to obtain data on the presence of infections (up to 6 months after initial blood sampling). Electronic drug charts were reviewed for evidence of antibiotic use. CXR, chest X-ray.

### 2.2 Functional analysis

In order to determine differences between immature PMN populations in patients with ARLD, whole blood was incubated for 30 mins with 2% dextran to allow erythrocyte sedimentation. The buffy coat was removed and layered on a density gradient of 56% and 80% Percoll. After centrifugation (220 x G for 20 minutes without brake), cells from either the upper (PBMC) or the lower (granulocyte) interface were removed and washed with RPMI-1640 + 1% PSG. Samples were then resuspended in 1ml HbSS and run on the Sysmex-XN 1000 analyser to determine differential cell counts. Neutrophil phagocytosis in heparin anticoagulated whole blood was assessed cytometrically using a PHAGOTEST kit (BD Biosciences, Oxford, UK) according to manufacturer’s instructions. For NETosis assays, neutrophils were isolated using density gradient centrifugation and 200,000 cells per well were incubated with either 25nM PMA or vehicle control (DMSO). After incubation for 3 hours at 37^ο^C, supernatant containing extruded nucleic acids was centrifuged at high speed (2,200G, 4^ο^C, 10mins) transferred black, flat-bottomed, polystyrene, 96 well plates and labelled with 1µM SYTOX green dye (Invitrogen, S7020). Fluorescence was quantified using excitation 485nm and emission 530nm (BioTek-Synergy 2 plate reader). Values were compared with a standard curve of purified lambda DNA (Thermo Fisher Scientific, SD0011) to quantify the degree of NET production as described by Hazeldine et al. (19).

### 2.3 Statistical analysis

Graphical data are illustrated with individual symbols representing each patient with mean values for the cohort indicated. Tabulated data includes median and interquartile range for the cohort. Data were reviewed for normality (using the Shapiro Wilkes Test), and depending on the result, they were analysed using a one-way ANOVA or Kruskal-Wallace test with multiple comparisons. Correlations were calculated using the Spearman’s rank correlation co-efficient.

## 3 Results

In this study we wished to understand the changes in peripheral blood myeloid populations which accompany alcohol-related liver disease with a view to identification of simply measured prognostic markers. Healthy volunteers were also used as a control cohort and although we aimed to match for donor age, our cohort was on average significantly younger than our patient groups (**Table 1**). Patients were composed of those recently admitted for AH, and abstinent patients with ARLD. In addition, a small number of our ARLD patients attending high dependency clinics were not abstinent (ARLD-Drinker). The AH patient group exhibited heterogeneity across multiple parameters on a par with the varied extent of drinking and underlying liver disease. For those patients whom we had the appropriate clinical parameters we calculated a FIB-4 score (20) as a simple noninvasive guide to likely fibrosis stage. This confirmed that the AH cohort had significantly higher FIB-4 scores than the ARLD patients (abstinent and drinkers). 89% of the AH patients had scores suggestive of an ISHAK fibrosis stage greater than 4, whilst 30% of drinking and 13% of abstinent patients fell into this category. The drinking patients with ARLD reported an average weekly alcohol unit consumption around 25% of that reported in the AH group but were a useful group to allow us to assess potential impact of alcohol superimposed on established disease given their consumption was over twice the recommended UK maximum weekly units. The AH patients exhibited significantly elevated MELD and Child Pugh scores compared to both ARLD groups (p<0.0001 for both), but MELD scores were similar across the ARLD groups. Decompensated liver function (evidenced by a MELD score >15) was observed in 95% of the AH patients whilst all the patients who were still drinking exhibited compensated liver function at time of assessment. In contrast nearly 20% of the abstinent ARLD patients had decompensated. The mortality risk was significantly elevated in the AH group as indicated by their elevated UKELD scores, and these patients had Maddrey’s scores above 50 on average. **Table 1** shows that these patients had an approximately 20% mortality risk at one month and this increased to around 30% at six months and to >40% at one year post initial presentation.

**Figure 1:**
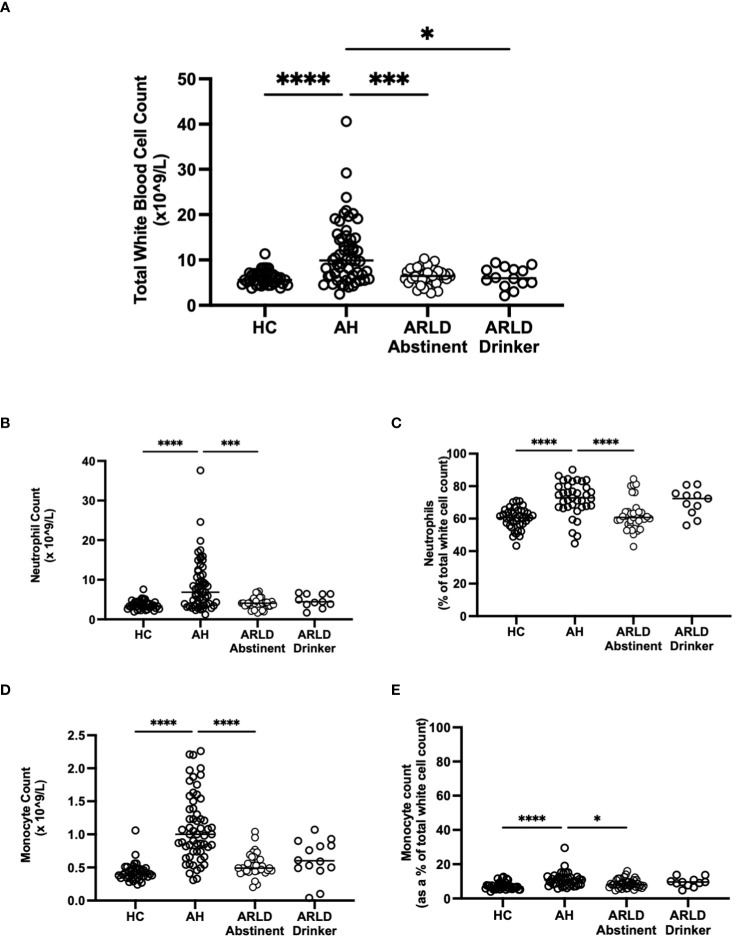
**(A)** Total white blood cell counts in whole blood samples obtained from patients with alcoholic hepatitis (AH), patients with alcohol related liver disease who were abstinent of alcohol, patients with alcohol related liver disease and ongoing drinking (Drinker), and healthy controls (HC), as measured by the Sysmex XN1000 analyser. **(B–E)** Neutrophil and Monocyte counts shown as total cell count or as a percentage of total WBC count as indicated. Individual data points represent a single patient, and the line is mean for the cohort. Multivariate ANOVA (Kruskal Wallis with Dunns correction) indicated significant differences between cohorts as indicated (p<0.05*, 0.01**, 0.001*** and 0.0001****).

**Figure 2:**
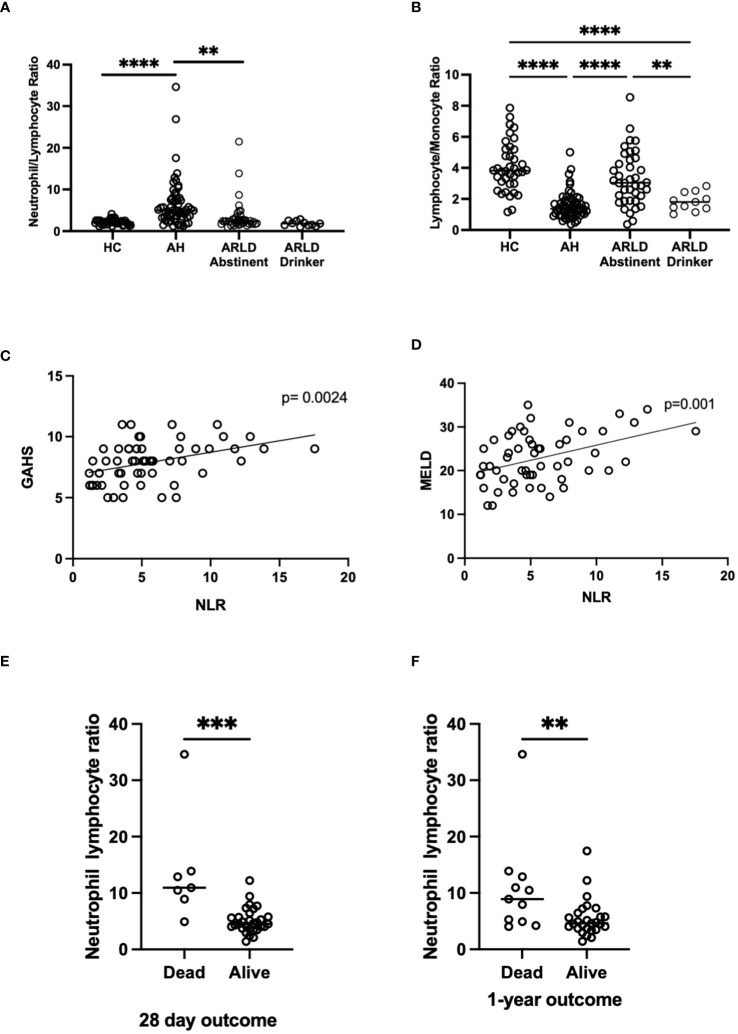
**(A, B)** Neutrophil: Lymphocyte, and Lymphocyte: Monocyte ratios in peripheral blood of patients with alcohol-related hepatitis (AH), patients with alcohol related liver disease who were abstinent of alcohol (ARLD-A) or still drinking (ARLD-D) and healthy controls (HC). **(C–E)** Mortality and infection data along with MELD, GAHS and biochemical characteristics were collected from electronic patient records of individuals with alcohol-related hepatitis. **(C, D)** Pearson r correlation analysis between NLR and GAHS or MELD score. Individual data points represent a single patient, and the line is best fit for the cohort. **(E, F)** NLR and survival data, multivariate ANOVA (Kruskal Wallis with Dunns correction) indicated significant differences between cohorts as indicated (p<0.05*, 0.01**, 0.001***, 0.0001****).

Patients with alcohol-related liver disease had elevated peripheral blood white cell count compared to healthy controls and this was particularly marked in those patients with alcohol-related hepatitis (**Figure 1A**). Here levels in AH significantly exceeded total white cell counts in abstinent ARLD and patients who were still drinking. Importantly neutrophils accounted for greater than 60% of the white cells in all individuals, with the largest proportion again in those individuals with AH (**Figures 1B, C**). Whilst the neutrophil percentage was consistently elevated in patients with AH, the neutrophil count showed significant interindividual variation ranging from (1.2 to 37.6 x10^9^/L, **Figure 1B**). This correlated to a certain extent with disease severity since those patients with the highest Glasgow Alcoholic Hepatitis Score (GAHS) score/Maddrey function also tended to have the most increased peripheral neutrophil count (**Supplementary Figure 2**). The same was true for circulating monocytes with both % monocytes and total count significantly elevated in AH compared to healthy controls (**Figures 1D, E**) and increased compared to abstinent and drinking individuals with alcohol-related cirrhosis. These observations are in keeping with reported changes in neutrophil: lymphocyte ratio (NLR) (16) and monocyte ratio (21) in AH. In agreement, we confirmed that NLR was significantly elevated in both AH and ARLD (**Figure 2A**) and lymphocyte: monocyte ratio (LMR, **Figure 2B**) was significantly reduced in all our patient groups. This was associated with patient outcome parameters, with NLR in AH correlated with both GAHS (**Figure 2C**), MELD score (**Figure 2D**) and renal function (Creatine, **Supplementary Figure 1**). Similarly, when we compared survival in our AH cohort, both 28 day and 1 year survival were significantly reduced in patients with elevated NLR (**Figures 2E, F**).

**Figure 3:**
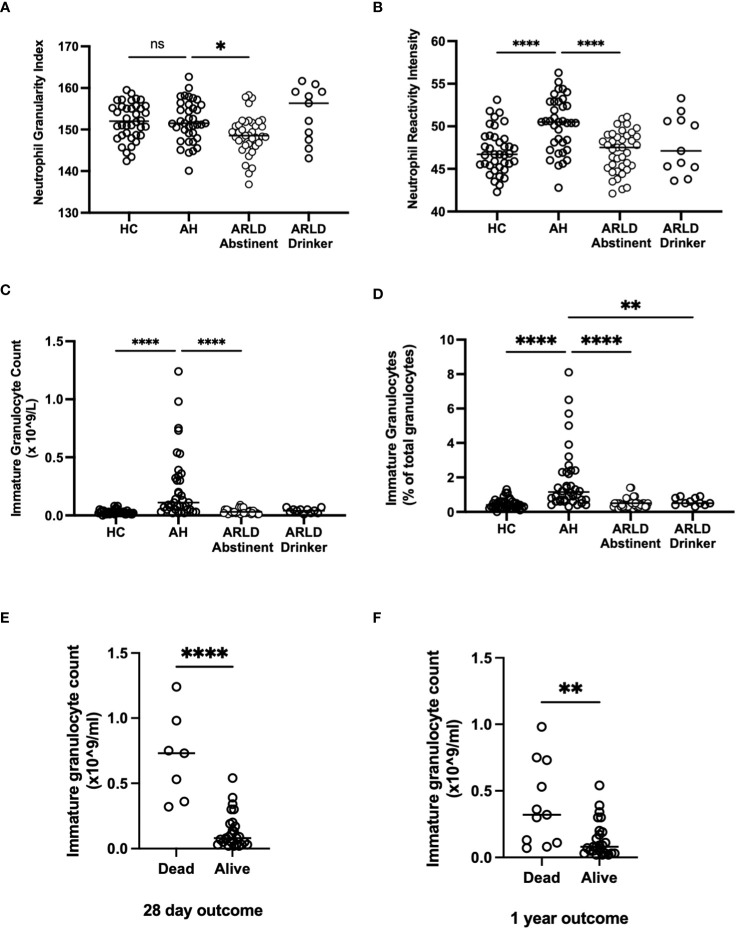
**(A, B)** Neutrophil granularity and reactivity intensity, **(C, D)** Immature granulocyte parameters and **(E, F)** Outcome data as assessed by Sysmex XN1000 analyser and extraction of data from electronic patient records. Values in peripheral blood from patients with alcohol-related hepatitis (AH), patients with alcohol related liver disease who were abstinent of alcohol (ARLD-A) or still drinking (ARLD-D) and healthy controls (HC). Mortality data was collected from electronic patient records of individuals with alcoholic hepatitis. Individual data points represent a single patient, and the line is mean for the cohort. Multivariate ANOVA (Kruskal Wallis with Dunns correction) indicated significant differences between cohorts as indicated (p<0.05*, 0.01**, 0.0001****) non-significant pairings are not annotated.

The Sysmex analyser permits assessment of key features of myeloid cell morphology such as cytoplasmic granularity and membrane fluorescence which can indicate functional status and maturity (22). **Figure 3** shows the data for cytoplasmic granularity (NEUT-GI) and reactivity intensity (NEUT-RI) for peripheral blood neutrophils in patients with AH compared to healthy controls and patients with chronic disease. Patients with AH had more reactive neutrophils than both healthy controls and abstinent ARLD patients (**Figure 3B**). The number of patients in our drinking ARLD group was relatively small in comparison but these patients again tended to have higher numbers of more granular neutrophils compared to the abstinent group. However, when immature neutrophils were compared, it was clear that these were significantly elevated only in the context of alcohol-related hepatitis and not in patients with chronic ARLD (even those still drinking, **Figures 3C, D**). This was an important distinction since the percentage of immature granulocytes in the peripheral blood population was associated with mortality outcomes. **Figure 3E** shows that there was a significant increase in the number of immature granulocytes in patients who died within 28 days of initial presentation, and that the impact persisted up to a year (panel **F**). Of note if we considered that patients with the top three highest NEUT-GI counts, this group all died by day 28, had a mean NEUT- GI of 154.8 and a mean NEUT-RI of 52.6. They also had an average GAHS score of 10.6 and were thus severely ill patients. Immature granulocytes have been reported previously to have altered immunosuppressive, phagocytic and metabolic functions (23, 24) and are found in elevated numbers in the blood of patients with conditions such as sepsis (23). These cells exhibit altered nuclear morphology and granule contents meaning they can be selected using differential centrifugation techniques. We were able to isolate and count cells from a small number of patients and we did indeed see elevated numbers of LD PMN separating into the lymphocyte band in AH compared to healthy controls (4.95 ± 0.6% of LD cells in AH vs 1.3 ± 0.4% in healthy controls). We also noted that isolated neutrophils from patients with AH had a reduced ability to exteriorize NETS in response to PMA compared to both healthy controls and patients with ARLD (**Supplementary Figure 3A**). In AH neutrophil phagocytic response was also blunted compared to healthy controls (**Supplementary Figure 3B**), although this response was not significant in the small number of samples available. This is in keeping with reports of neutrophil dysfunction in other acute inflammatory settings (25, 26).

**Figure 4:**
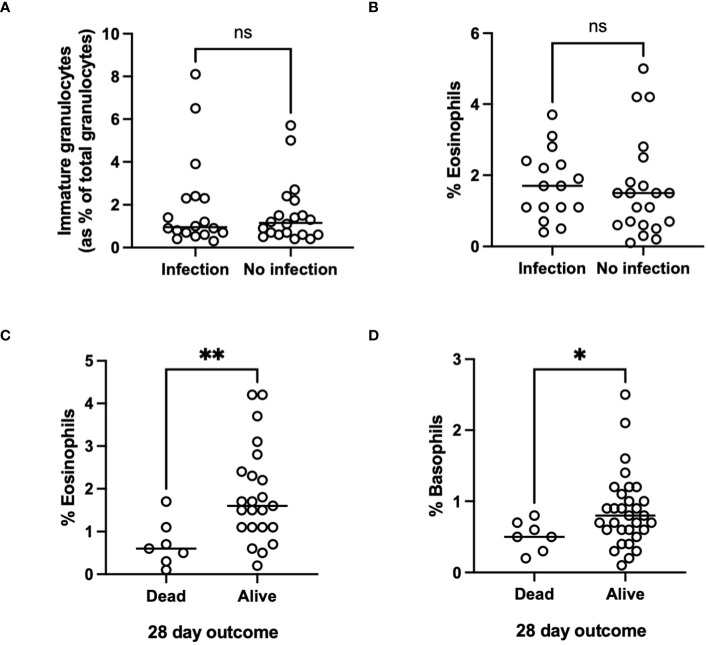
**(A, B)** Infectious outcome, or **(C, D)** Mortality at 28 days and 1 year in patients with alcohol-related hepatitis. Data was collected fusing Sysmex XN1000 analyser or from electronic patient records and individual data points represent a single patient, with the line indicating mean for the cohort. ANOVA indicated differences between cohorts as indicated (p<0.05*). p<0.01 **, ns, indicates no significant difference between indicated cohorts.

Although the proportion of AH patients in whom we could document evidence of infection (**Table 2**) was relatively low in our cohort we did observe respiratory infections and spontaneous bacterial peritonitis in the alcoholic hepatitis cohort with a total of 40/58 patients being administered antibiotics (**Table 2**). Those patients with the highest immature neutrophil counts had increased risk of developing infection (**Figure 4A**). Interestingly this held true when we considered some of the rarer granulocyte populations (eosinophils and basophils) too. Eosinophil number was variable in the cohorts (Healthy control: 0.15 ±0.015 x10^9^/L, AH: 0.154 ±0.02 x10^9^/L, ALD abstinent: 0.2 ±0.041 x10^9^/L, ALD Drinker: 0.12 ±0.025 x10^9^/L) but when the cell count was considered as a percentage of total WBC count, there was a significant increase in eosinophil % in those individuals who were alive at 28 days (**Figure 4C**). Again, there was a tendency for higher eosinophil counts in those patients who did not show signs of infection, but this was not significant (**Figure 4B**). A similar pattern was apparent for basophils where again individual counts varied across the groups but were reduced in all patients vs healthy controls (Healthy control: 0.36 ±0.16 x10^9^/L, AH: 0.07 ±0.006 x10^9^/L, ALD abstinent: 0.15 ±0.010 x10^9^/L, ALD Drinker: 0.048 ±0.006 x10^9^/L). Here, increased basophils as a percentage of WBC were observed in those patients who were alive at 28 days (**Figure 4D**).

## 4 Discussion

In the present study we utilized peripheral blood from cohorts of patients across the spectrum of ARLD to document changes in peripheral myeloid cells populations. We include data from patients with ARLD that are abstinent (ARLD-A) and patients with ARLD with ongoing alcohol use (ARLD-D). This permits consideration of whether differential findings observed in the AH patients simply due to the presence of chronic disease or alcohol alone. We confirmed that total white cell number is increased in acute alcohol-related hepatitis but not drinking or abstinent ARLD, and that elevation of myeloid cells in particular (both neutrophils and monocytes) may underlie this observation. These findings are in keeping with previously published work (16, 21), which reassures that our inclusion criteria and disease severity for the AH group are similar to those used in other settings (27). However, the variation within our acute alcohol group (29/38 patients having a Maddrey DF>32, and 9/38 a Maddrey DF≤32) likely explains the spread of data for white cell parameters in this cohort, even though 95% of patients had a MELD> 15. It has been suggested that inflammation causes reciprocal production of granulocytes and lymphocytes in the bone marrow (28). Both cell types develop within the same niche, and therefore compete for resources. Inflammation associated reductions in growth and retention factors, such as stem cell factor and CXCL12, which act preferentially to inhibit lymphocyte development may explain the relative reductions in peripheral lymphocyte number (28). There is published data showing that neutrophil: lymphocyte ratios are elevated in patients with AH, and when incorporated within the GAHS score, can enhance the accuracy of the GAHS score in predicting mortality in AH (16). Similarly, data in cirrhotic patients listed for liver transplantation suggest that elevated NLR predicts poor outcome (29). Thus, it is interesting to observe that our drinking ARLD patients who were not listed for transplant and had higher average MELD score than the abstinent group, also had a higher NLR. We also report that those patients with the lowest NLR in AH had the best mortality outcomes at both 28 days and 1 year. Certainly, our data correlating NLR with both GAHS and renal function in the AH cohort are in keeping with these findings (16).

Circulating monocyte counts were also elevated particularly in the AH group compared to healthy controls and abstinent patients. Others using similar analytical approaches have suggested that monocyte distribution width (MDW, an indicator of cell activation) increases in response to infection and is an indicator of suspected sepsis (30). We were unable to show an association with infection in our relatively small cohort (although peripheral monocyte count was highest in those without sign of infection) and thus it would be interesting to explore MDW in future larger studies.

Perhaps the most significant and novel observation in our study was the presence of significant numbers of immature granulocytes in the peripheral blood of patients with alcohol-related hepatitis. The Sysmex analyser identifies these cells (NEUT-IG) based on both their cytoplasmic granular complexity and abundance of nucleic acids detected by fluorescent staining (31). The identified cells are typically myelocytes and there is a reported good correlation between the number of these cells as detected by the Sysmex instrument and traditional histopathological assessment of blood films (32). These correlate with disease severity (GAHS) and are associated with increased short-term mortality and likelihood of infection. The utility of the NEUT-IG parameter has been investigated for its diagnostic value in sepsis (26, 33–36) with the suggestion that emergency granulopoiesis and high frequencies of immature circulating cells are linked to poor outcome (37). Numbers of immature granulocytes are variable in the circulation of both healthy individuals and patients with inflammatory conditions, but there is a tendency for an increased numbers in disease (38). However an important caveat is that it has also been noted that in patients with some leukaemias or situations with elevated numbers of band neutrophils, the instrument may falsely elevate the IG count (39). One solution is to consider low density neutrophil (LDN) populations which have been suggested to represent immature neutrophil populations (23). We were able to use density gradient centrifugation to isolate low density neutrophils (LDN), and these were increased in AH. LDN have reduced ability to undergo phagocytosis, generate an oxidative burst and produce cytokines from whole blood. It is reported that these cells can include populations of more immature granulocytes, and that the extent of stimulated degranulation of neutrophils correlates with the number of low-density neutrophils present (23). However there is also evidence that some populations of LDN that separate within the lymphocytic cell fraction are actually a mature, primed cell population with enhanced ROS generation and phagocytic capacities (40). We were unable to purify sufficient LDN for detailed functional analysis, but we did note a tendency for reduced phagocytic capacity and stimulant liberated netosis of whole PMN populations from AH patient blood. Both would reduce the antimicrobial capacities of patient cells and have been linked to infection risk in other inflammatory contexts (25, 26). Our functional data would suggest that we are indeed considering immature cells as opposed to primed or ‘suppressor’ type cells (41), but further cytometric phenotyping of isolated cells would be necessary to absolutely confirm this (42).

It is currently unclear in the literature whether the absolute number of immature granulocytes versus the percentage of immature granulocytes provides more useful information. We found that absolute counts of immature granulocytes were more closely associated with disease severity and mortality than percentages. Whilst it is possible that the raised immature granulocyte count in AH is simply a marker of bone marrow stimulation, it is also feasible that the increased counts of immature granulocytes, which are less functional, contribute to the observed neutrophil dysfunction and risk of sepsis in AH (43, 44). Our total immature cell count was a relatively small proportion of total neutrophils but combined with reductions in both eosinophils and basophils could contribute to impaired pathogen clearance and thus explain our survival data. Our data also suggests a trend for worse infectious outcomes in patients with the lowest eosinophil and basophil number. Eosinophil number, and in particular eosinophil lymphocyte ratio has been considered as a marker of alcohol use in patients with bipolar disorder (45) may relate to increased IL-5 and TNF levels, and production and release of eosinophils from bone marrow (46). Recruitment of eosinophils to the liver is a protective feature in the context of acute hepatitis (47), so it is interesting to speculate whether patients with increased eosinophil release to the periphery may also show increased hepatic recruitment.

Of note along with an increase in immature neutrophils, we also demonstrated increased numbers of more granular and ‘reactive’ neutrophils (48) in alcoholic hepatitis and in the case of granularity this was also observed in our ARLD drinking cohort. Here the sysmex analyser measures relative membrane fluorescence (Neut-RI) along with cytoplasmic complexity and granule composition via 90° light scatted (NEUT-GI) to give an indication of proportions of more metabolically activated cells. These tend to be considered as more ‘activated’ and so our findings are in keeping with reports that patients with AH had increased neutrophil activation in comparison to healthy volunteers, as demonstrated by loss of CD62L expression and increased basal oxidant production (49). Similar populations have been described in patients with autoimmune hepatitis (48), but we believe we are the first to use the Sysmex system to describe these more reactive cells in drinking patients with chronic ARLD and AH. Further consideration of the more unusual granulocyte populations (basophils and eosinophils) is also warranted based on our preliminary findings. Pro-inflammatory ‘low density’ neutrophils have been described in cirrhosis (50) and contribute to organ damage upon recruitment into tissue. These ‘primed’ cells are suggested by some to have impaired ROS production and compromised bacterial killing ability, although findings vary depending on the nature and severity of cirrhosis (50). Combined with reported deficiencies in neutrophil chemotaxis (51) and altered NET formation (52) in cirrhosis it is easy to appreciate how predisposition to bacterial infections may occur. However, we should note that our Unit has a cautious approach to initiation of antimicrobial therapy and thus overall, our reported incidence of, and mortality from infection was low. Similarly, infections which presented after discharge or patients who subsequently presented to other Centers would not feature in our data set. Thus, we may be underestimating the true incidence of infections in our study. Despite this and the relatively small size of our cohorts we believe there is potential to measure immature granulocyte counts as a simply calculated predictor of mortality and risk of infectious complications in alcohol related disease. This would allow identification of patients who may require more intensive management. Our study is novel in that we have tried to consider the full spectrum of alcohol related disease in humans from alcohol-related hepatitis to cirrhosis and have utilized a simple approach that translates to most clinical settings. A major advantage of using an automated hematology analyzer to obtain information on myeloid parameters is that no additional processing steps are required between obtaining the sample and running it on the analyser, which minimises the risk of inadvertent neutrophil activation during isolation/processing steps. Thus, in conclusion we suggest that measurement of myeloid parameters such as immature granulocyte count has potential prognostic ability in alcohol-related liver disease.

## Data availability statement

Anonymised raw data supporting the conclusions of this article may be made available on personal request to the authors.

## Ethics statement

The studies involving humans were approved by Wales Research Ethics Comittee (REC ref 18/WA/0214). The studies were conducted in accordance with the local legislation and institutional requirements. The participants provided their written informed consent to participate in this study.

## Author contributions

RK: Data curation, Formal analysis, Investigation, Methodology, Writing – original draft. SS: Data curation, Formal analysis, Investigation, Writing – review & editing. LH: Data curation, Investigation, Writing – review & editing. LS: Data curation, Formal analysis, Methodology, Writing – review & editing. JH: Methodology, Writing – review & editing. NR: Writing – review & editing. PN: Funding acquisition, Supervision, Writing – review & editing. PL: Conceptualization, Data curation, Formal analysis, Funding acquisition, Supervision, Writing – original draft, Writing – review & editing.
