# Supplementary figures and images for: Circulating myeloid populations have prognostic utility in alcohol-related liver disease
Source: Front Immunol. 2024 Mar 13;15:1330536. doi: 10.3389/fimmu.2024.1330536 (PMC10965684; doi:10.3389/fimmu.2024.1330536)

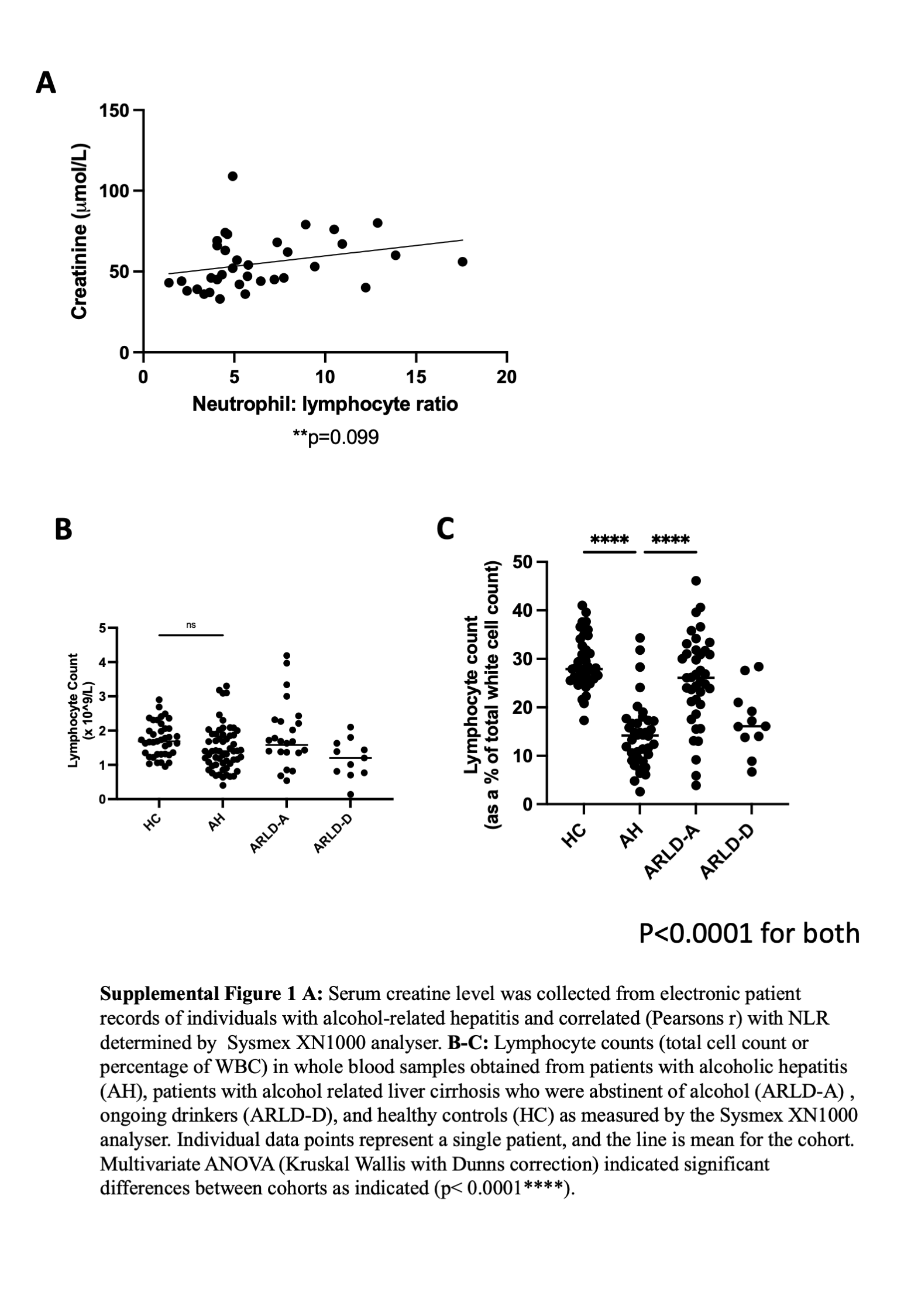

Supplement: Supplementary file 1 [file Image_1.tiff]

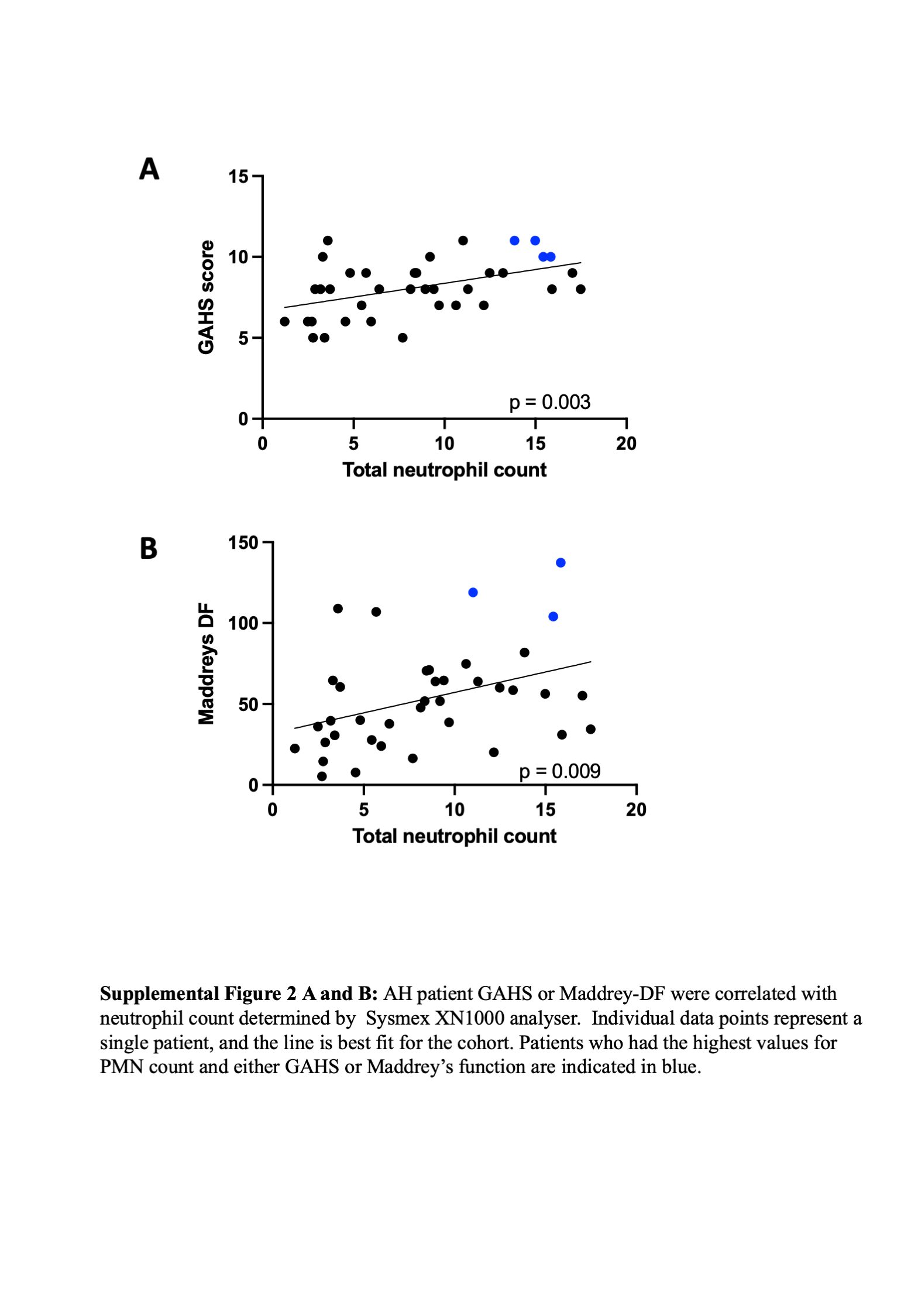

Supplement: Supplementary file 2 [file Image_2.tiff]

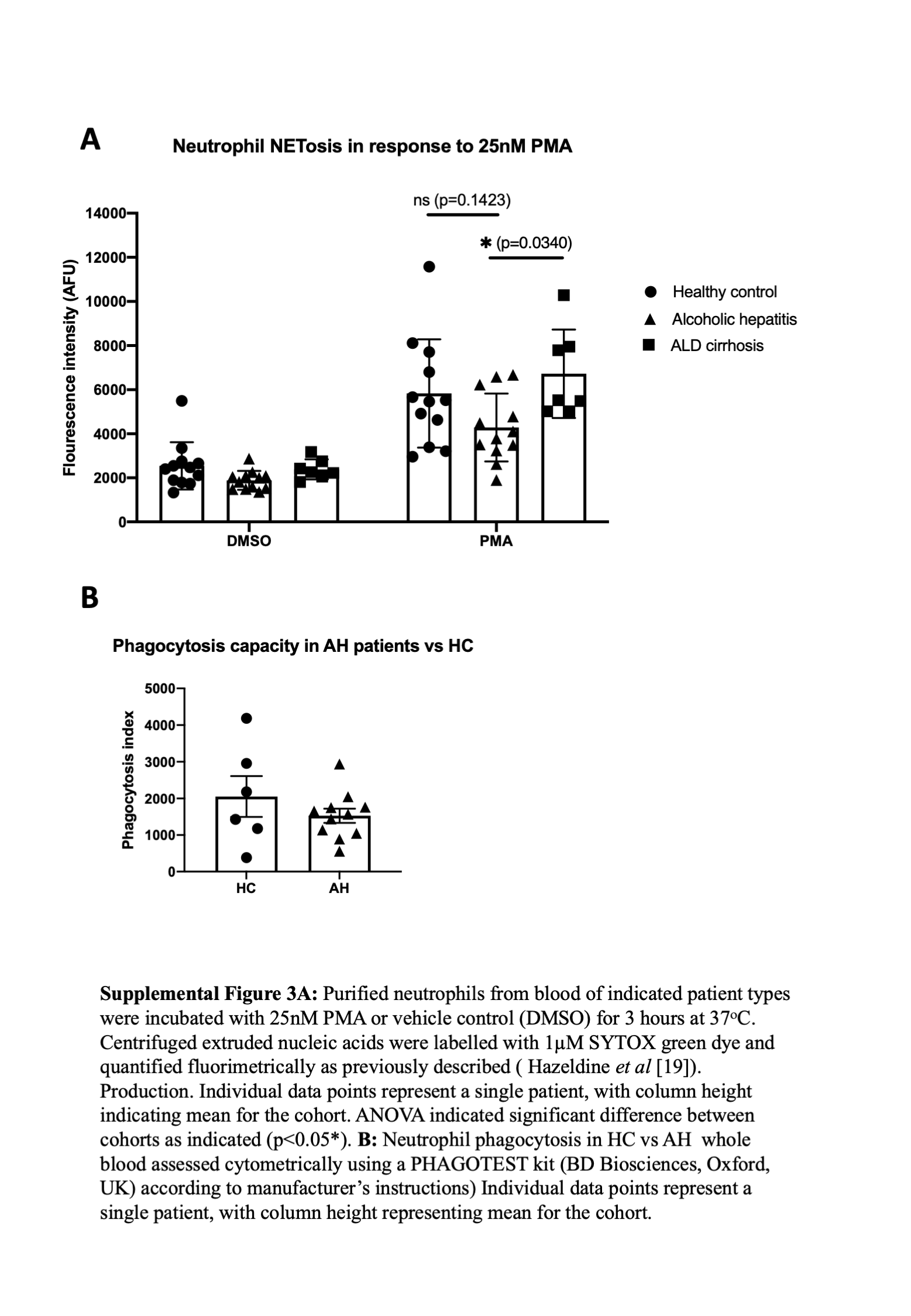

Supplement: Supplementary file 3 [file Image_3.tiff]
